# A new anterior approach to parastomal hernia repair (PHR) with linear stapler suture: A case report

**DOI:** 10.1016/j.amsu.2020.11.038

**Published:** 2020-11-17

**Authors:** Serra Francesco, Isabella Bonaduce, Francesca Cabry, Lorena Sorrentino, Tommaso Iaquinta, Sara Fenocchi, Gelmini Roberta

**Affiliations:** Department of Surgery, University of Modena and Reggio Emilia – Policlinico of Modena, Modena Italy, Via del Pozzo, 71 41100, Modena, Italy

**Keywords:** Parastomal hernia, Anterior approach, Linear stapler suture, Case report

## Abstract

**Introduction:**

Parastomal hernia is a type of incisional hernia occurring in abdominal integuments in the vicinity of a stoma. The best surgical approach for PSH remains controversial. Most studies report short follow-up time after surgery and a low number of cases to allow conclusions. Actually, we don't have a relevant recommendation about an optimal surgical technique or the most effective mesh for PSH repair.

**Presentation of the case:**

Once packaged the latero-lateral mechanical anastomosis to restore the continuity of the intestinal tract of the patient, an adequate disinfection of trough of the stoma was done. The lateral and medial margins of the defect are then transposed towards each other and kept side by side with a gripper; a 60 mm tristaple linear stapler was placed, incorporating both edges and the charge is fired to obtain a perfect synthesis of the retromuscular plane.

**Discussion:**

In the literature has been described several surgical techniques for its repair: suture repair, relocation, mesh-based technique with open or laparoscopic approach. Both, the simple corrective surgery of Thorlakson in 1965 and the use of the peritoneomuscular flap for closing the defect, suggested by Bewes, led to high incidence of recurrence. An important reduction in the rate of parastomal hernia derives also from the mesh reinforcement of the stoma trephine.

**Conclusion:**

The authors suggest that this technique should be help the surgeons to repair parastomal hernia in patients with multiple risk factors to develop a recurrence of parastomal hernia.

## Highlights

• The best surgical approach for Parastomal Hernia repair (PSH) remains controversial.
• Causes that predispose to the development of incisional hernia are multiple, such as obesity and diabetes.
• This technique may help the surgeons to repair the PSH in patients with risk factors to develop a recurrence after surgery.

## 1 Introduction

Parastomal hernia is a type of incisional hernia occurring in abdominal integuments in the vicinity of a stoma [2]. It is a common complication following the creation of an intestinal stoma, with an incidence of 58% in systematic reviews [3], which can cause discomfort, pain, bowel strangulation and incarceration as well as difficulties with stoma care [4].

The best surgical approach for PSH remains controversial. Most studies report short follow-up time after surgery and low number of cases to allow conclusions. Evidence provided by retrospective case series suggests that biological meshes are associated with high recurrence rates and may demonstrate some benefit in terms of mesh infection [5,6].

The literature on the safety of combining stoma reversal with an additional procedure, such as the parastomal hernia repair, is limited. The optimized time and the cost-effectiveness could make this procedure tempting, even if only a few of study found lower cost of a single-stage technique. A combined approach induces a long operative time and important perioperative stress. Moreover the risk of complications after exposure to two separate surgical and anesthesiologic procedures are significant [7]. Several authors discussed about the risks associated performing ventral hernia repair with other abdominal procedure concomitantly. The study of Maggiori et al. [8] reported a wound infection rate of 7% and an important lesser rate of hernia occurrence at one year in patients undergoing ileostomy closure with a reinforce through retromuscular biological mesh [8]. About the surgical technique only a few of study discussed the concomitant hernia repair and stoma closure. Keisuke et al. [9] described a surgical approach in which they used the bilateral anterior rectus abdominis sheath turnover flap method, avoiding to use a mesh for the risk of infection. It seems to be useful for patients with large incisional hernia [9].

Causes that predispose to the development of incisional hernia are, in addition to the presence of the stoma which is itself a factor of weakening of the wall, the obesity, the diabetes, the concomitance of chemotherapy in patients who have a terminal colostomy for neoplasia of the colon.

Carraro et [10]. Al have described good results using linear stapler to repair the abdominal midline weakness.

Actually, we don't have relevant recommendation about an optimal surgical technique or the most effective mesh for PSH repair.

After this consideration, we have considered to repair a PHR about 8 cm of diameter, in an obese patient (BMI 34.8), diabetic and carrier of ileostomy.

Evaluate the comorbidities of the patient as well as the BMI, we decided to repair the defect using a linear stapler to reconstruct the posterior fascia under rectum muscle and apply a resorbable mesh with a resorbable hydrogel coating mesh in sublay position. This paper aims to share a new surgical technique because the authors believe that in patients with multiple risk factors of developing a recurrence of parastomal hernia, on a dirty surgical site, a triple layer mechanical suture can equalize the traction forces on the suture, reducing the risk of tearing the fascia itself.

## 2 Presentation of the case

A 60 years-old Caucasian man came to our observation to treat a voluminous parastomal hernia. The patient's medical history was positive for diabetes mellitus (DM type 2) insulin-independent, metabolic syndrome (BMI 34.8), and was subjected to appendectomy in 1982.

**Fig. 1:**
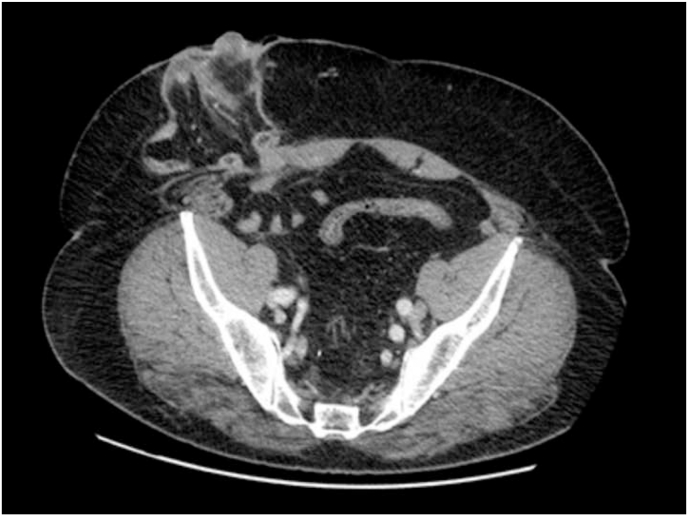
Pre-operative ct-scan for the study of the parastomal hernia.

In 2019 he was diagnosed with rectal cancer; then was undergone surgery that consisted of laparoscopic rectal resection and packaging of loop ileostomy. After the surgery, the patient has been treated with adjuvant chemotherapy according to the Xelox scheme (8 cycles). After one year of surgery, he developed a voluminous parastomal hernia that was studied with a CT-Scan; after the visualization of the imaging, was given the indication to close the ileostomy and repair the hernia. The surgical procedures was conducted by 2 well-trained surgeons in wall repair. Fig. 1

Once packaged the latero-lateral mechanical anastomosis to restore the continuity of the patient's intestinal tract, adequate disinfection of the trough of the stoma was done.

Then, appropriate preparation of the peritoneal plane below the right rectum muscle was performed.

The defect's lateral and medial margins are then transposed towards each other and kept side by side with a gripper; a 60 mm tristaple linear stapler was placed, incorporating both edges and the charge is fired to obtain a perfect synthesis of the retromuscular plane.

**Fig. 2:**
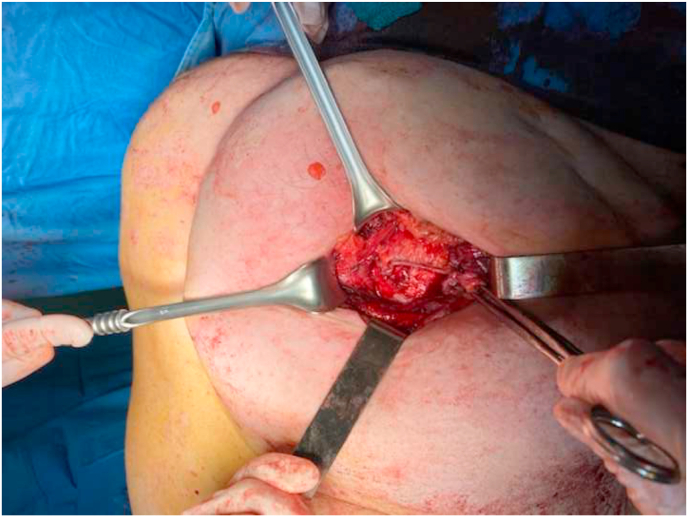
The posterior fascial plan is completely closed by linear stapler suture.

A second charge is applied to terminate the closure of the defect. Fig. 2

**Fig. 3:**
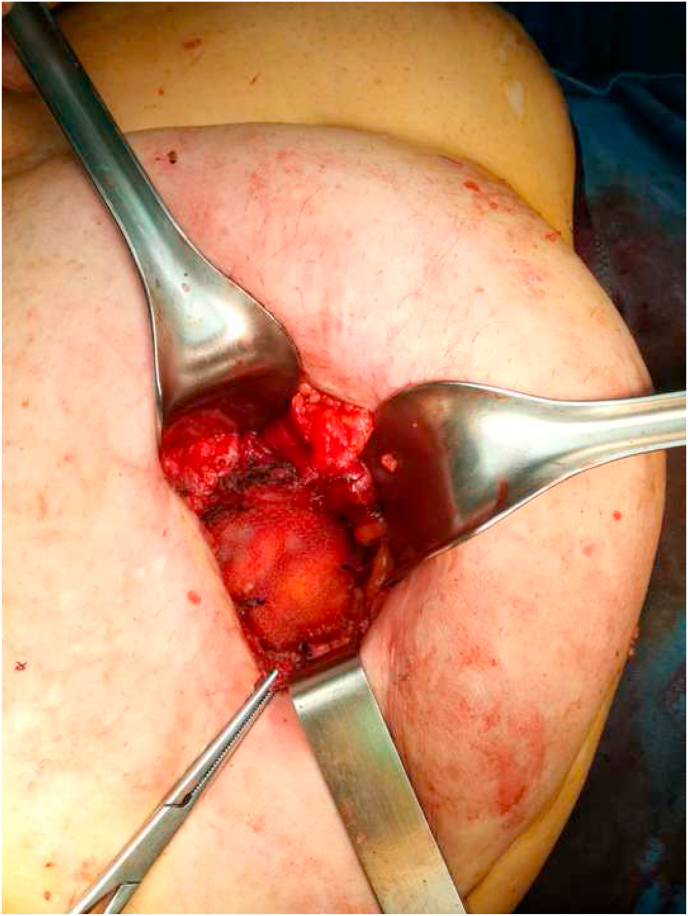
A resorbable mesh with a resorbable hydrogel coating was placed in sub-lay space.

A resorbable mesh with a resorbable hydrogel coating was placed in sub-lay space, to reduce the risk of infection of the surgical site (SSI)and was fixed with resorbable points. Fig. 3

The anterior fascia of the right muscles is synthesized in detached points.

**Fig. 4:**
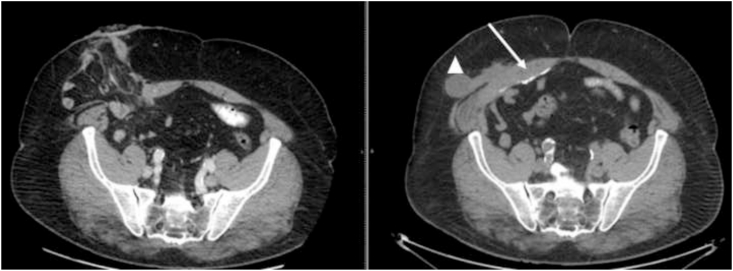
A comparison between the pre-operative CT-scan and that performed after 5 months from surgery: the row indicate the complete repair of the hernia. A triangle indicate a small seroma about 2 cm that is now reabsorbed.

No pain, no SSI was registered during the hospitalization. The patient was discharged in 12th post-operative day. After discharge, the patient performed the ambulatory control for 2 weeks, 1 time a week. After 5 months from surgery a CT-scan was performed to confirm that, to date, there is no evidence of recurrence of the hernia. Fig. 4.

The mesh used is a resorbable monofilament of poly-4-hydroxybuterate (P4HB) which has been indicated in the repair of hernias of obese and diabetic patients; furthermore, this material should reduce the risk of infection of surgical site [11]. After 18 months the mesh is perfectly integrated into the patient's wall, removing the infectious risk of a non-absorbable mesh. The authors believe that the application of a resorbable mesh is indicated in case of optimal reconstruction of the posterior fascial plane, which we have tried to achieve by using the tristaple linear stapler.

## 3 Discussion

Considering the good results that some authors have obtained with applying the linear stapler to repair the incisional hernia, we have applied the same concept to repair the PSH. The main challenge encountered in applying this technique is the lack of healthy tissue; in this case it was necessary to make small incisions at the proximal and distal margin of the solution of continuity to make it oval-shaped; subsequently the fascial plane was isolated until an adequate overlap was obtained to correctly apply the stapler. At this stage, extreme caution is essential to avoid to pinch a small bowel in the suture.

The literature has described several surgical techniques for its repair: suture repair, relocation, mesh-based technique with open or laparoscopic approach. Both, the simple corrective surgery of Thorlakson in 1965 and the use of the peritoneomuscular flap for closing the defect, suggested by Bewes, led to high incidence of recurrence. The relocation approach resulted also unsuccessful due to the herniation at the new stoma site, as well as at the original gap. These outcomes were based on the fact that parastomal and incision hernias represent a biologic disease rather than a simple mechanical rupture [2,12].

The first to publish findings on the use of prosthetic reinforcement for PSH repair were Hopkins and Trento [13]. They suggested that patient with recurrent hernia formation, following multiple abdominal procedures, was the ideal candidate for the use of a mesh [13]. At first, the idea of absorbable materials in contact with organs and the placement of an implant in the contaminated stoma environment led to the conclusion that the use of mesh for the repair of PSH was not advisable. Afterwards, with the development of biomaterials, which are better integrated and cause a lower inflammatory response, the technique gradually became the gold standard in correcting parastomal hernia [14]. Actually, the most representative procedure for surgical repair is the Sugarbaker technique which consist on the preparation of the hernial sac after laparotomy, lateralization of the colon, and placement of a prosthetic mesh in intraperitoneal onlay position (IPOM) [15]. The repair of hernia depends quite completely on the presence of an adequate positioned mesh and a material highly resistant to bacterial infections, as it follows from the study of Hansson et al. [16] They used the ePTFE (Expanded polytetrafluoroethylene), for its inert, soft and pliable material, even if the tendency to shrink of that material led to an high rate of recurrence [16].

In addition, some studies like that of Szczepkowski et al. [17], about the HyPER technique (hybrid parastomal endoscopic re-do), a combination of the laparoscopic and open approach for the PSH repair, stressed that the removal of the hernia sac, open or laparoscopic, and the creation of a correct diameter of the ostomy canal represent the most important goal for the PSH repair. Contrary to Le Blanc consideration [18] about the security of the repair, based on a patch size and adequate patch fixation rather than closure of the hernial orifice, some authors support the idea that the narrowing of the hernia orifice could seriously influence the recurrence rate.

An important reduction in the rate of parastomal hernia derives also from the mesh reinforcement of the stoma trephine. The difficulty to perform that laparoscopically led Williams et al. [19] to the creation of a new technique: the Stapled Mesh stoma Reinforcement Technique (SMART). It is a means of creating a reinforced stoma trephine combining open and laparoscopic surgery through the use of a stapler to create a circular trephine in the mesh, which was then sewn to the ventral aspect of the anterior rectus sheath as reinforcement [19].

Later, the group of Majumder et al. [20] suggested the Stapled Transabdominal Ostomy Reinforcement with Retromuscular Mesh (STORRM) with the aim of creating a technique that would result in a unified aperture of the optimal size. The use of an EEA (end-to-end anastomosis) stapler, in the STORRM, allows for the alignment of multiple layers creating consistently sized apertures. It ensures the aperture remains fixed despite patient movement and abdominal wall closure, also it decreases the chance for parastomal hernia recurrence through the ring of staples, which prevents radial expansion of the fenestrations along the axes of the cruciate incision. Unlike the SMART technique, the STORRM allows the creation of a unified aperture through anterior rectus sheath and large underlying mesh in one reinforced passage [20]. Zia et al. [21], instead, suggested a simple fascial suture laparoscopic repair of PSH by the use of the Crochet hook needle (EndoClose), with the result of reducing pain, lead to a shorter hospitalization, a lower risk of infection and, without the use of a mesh, a reduced risk of seroma formation [21].

Because of the lack of comparative evidence about different meshes for parastomal hernia repair, retrospective case series suggests that biological meshes are associated with high recurrence rates (ranging between 16 and 90%) and may demonstrate some benefit in terms of mesh infection [22]. In the study of Oma et al. [23] resulted a low risk of complications after parastomal hernia repair with Composite Parastomal Mesh for its semi-translucency which gives the surgeon a better overview. In addition the collagen-coated material on the visceral side of the mesh reduces the risk of intraperitoneal adhesions compared with uncoated mesh material [23].

Recently, three-dimensional (3D) funnel-shaped meshes have become available for the repair of PSH. These can give several advantages from the use in laparoscopic assisted and open surgery, to the locally coverage of the defect with a wide overlap to all sides and the use of a second flat mesh to treat concomitant incisional hernias. The dual-layer structure, with polyvinylidene fluoride and polypropylene, provides safe and rapid ingrowth in the abdominal wall. Moreover, the elasticity and flexibility of funnel mesh part fits the diverted bowel and protects against stoma prolapse. Complications such as hematoma and infections, compared with open technique, decrease since there isn't a separation of the different layers of the abdominal wall [24,25].

It is advisable to use a resorbable mesh only if the reconstruction of the posterior fascia is well-managed and tension-free; an under tension suture, that could occur if the abdominal wall is weak, is more likely to recurrence. In this case, is more appropriate to place over the suture a non-resorbable mesh.

## 4 Conclusion

To date, the parastomal is a challenging complication that surgeons have to face daily and there are no standardized methods for the repair of the defect. In this paper the authors described a technique to repair the PH in a patient with multiple risk factors to develop a recurrence of incisional hernia and SSI.

The defect can result in a functional alteration of the stoma as well as an aesthetic discomfort for the patient that can condition the sociability of the patient. This technique, using a linear stapler, is easy to replicate in the operative settings and permit to obtain an adequate reconstruction of the abdominal wall, which results in complete functional recovery, improving the quality of life of the patient itself. A large case series is necessary to standardize this technique.

## Ethical approval

No ethical approval was required.

## Sources of funding

Non funding were used.

## Author contribution

Serra Francesco, MD

Department of Surgery, University of Modena and Reggio Emilia – Policlinico of Modena, Modena Italy

Via del Pozzo, 71 41100 Modena Tel: +390594223662 FAX: +390594224370

serrafrancescomd@gmail.com

ORCID 0000-0002-2701-4387

Author and supervisor of entire manuscript

Isabella Bonaduce, MD

Department of Surgery, University of Modena and Reggio Emilia – Policlinico of Modena, Modena Italy

Via del Pozzo, 71 41100 Modena Tel: +390594223662 FAX: +390594224370

bellabonaduce@libero.it

Co-author of entire manuscript

Francesca Cabry, MD

Department of Surgery, University of Modena and Reggio Emilia – Policlinico of Modena, Modena Italy

Via del Pozzo, 71 41100 Modena Tel: +390594223662 FAX: +390594224370

francescacabry@gmail.com

Data collection and co-author of case report and discussion

Lorena Sorrentino, MD

Department of Surgery, University of Modena and Reggio Emilia – Policlinico of Modena, Modena Italy

Via del Pozzo, 71 41100 Modena Tel: +390594223662 FAX: +390594224370

lorena.sorrentino@live.it

Data and imaging collection

Tommaso Iaquinta, MD

Department of Surgery, University of Modena and Reggio Emilia – Policlinico of Modena, Modena Italy

Via del Pozzo, 71 41100 Modena Tel: +390594223662 FAX: +390594224370

Tom91@libero.it

Review of literature

Sara Fenocchi, MD

Department of Surgery, University of Modena and Reggio Emilia – Policlinico of Modena, Modena Italy

Via del Pozzo, 71 41100 Modena Tel: +390594223662 FAX: +390594224370

sarafenocchi@gmail.com

Review of literature and co-author of discussion

Gelmini Roberta, MD PhD

Department of Surgery, University of Modena and Reggio Emilia – Policlinico of Modena, Modena, Italy

Via del Pozzo, 71 41100 Modena Tel: +390594223662 FAX: +390594224370.

roberta.gelmini@unimore.it

ORCID 0000-0002-8471-710X

Supervisor and co-author of entire manuscript

## Research registration number

The submitted case report is not a research study.

## Guarantor

Serra Francesco, MD

Department of Surgery, University of Modena and Reggio Emilia – Policlinico of Modena, Modena Italy

Via del Pozzo, 71 41100 Modena Tel: +390594223662 FAX: +390594224370

serrafrancescomd@gmail.com

ORCID 0000-0002-2701-4387

## Provenance and peer review

Not commissioned, externally peer-reviewed.

## Disclosure statement

The authors have nothing to disclose.

The work was written in line with the SCARE criteria [1]

Written informed consent was obtained from the patient for publication of this case report and accompanying images. A copy of the written consent is available for review by the Editor-in-Chief of this journal on request.

## Declaration of competing interest

No conflicts of interest.
